#  Hepatoprotective properties of p-coumaric acid in a rat model of ischemia-reperfusion 

**Published:** 2020

**Authors:** Farkhondeh Parvizi, Parichehreh Yaghmaei, Seyed Ali Haeri Rohani, Seyed Ali Mard

**Affiliations:** 1 *Department of Biology, Faculty of Basic Science,* *Science and Research Branch, Islamic Azad University, Tehran, Iran*; 2 *Alimentary Tract Research Center, Physiology Research Center, Department of Physiology, School of Medicine, Ahvaz Jundishapur University of Medical Sciences, Ahvaz, Iran*

**Keywords:** p-coumaric acid, Antioxidant, ALT, SOD, Rat

## Abstract

**Objective:**

The liver as a highly metabolic organ, has a crucial role in human body. Its function is often impressed by changes of the blood flow, hypovolemic shock, transplantation, etc. Maintaining liver function is a major challenge and there are many approaches to potentiate this organ against different stresses. Antioxidants protect organs against oxidative stress. P-coumaric acid (PC) as an oxidant has many beneficial effects. Therefore, PC was used as a pretreatment to test its potential against oxidative stress induced by liver Ischemia-reperfusion injury in rats.

**Materials and Methods:**

In order to test the potential hepatoprotective effect of PC against IR injury, five groups of rats were used: Normal (NC; intact group); Sham; p-coumaric acid (PC); IR-CO, and PC-IR. PC, Sham, NC, PC-IR and IR-CO groups that received vehicle or p-coumaric acid at a dose of 100 mg/kg for 7 consecutive days as pretreatment before IR induction. Animals in PC-IR, and IR-CO groups underwent hepatic IR injury. Liver levels of antioxidants were determined and functional liver tests were done. Hematoxylin and eosin staining was done to determine the structural changes of the liver. Gene expression of caspase-3 was also assessed.

**Results:**

Hepatic IR injury disrupted liver function by increasing the levels of AST, and ALT, and decreasing GSH, SOD and catalase. PC significantly decreased liver inflammation, reverted liver functional enzymes and antioxidants levels to normal, reduced the gene expression of caspase-3 in PC-IR rats compared to the IR-CO group.

**Conclusion:**

These findings revealed that PC through improving liver´s antioxidants, liver functional tests and down-regulating apoptotic gene protein, caspase-3, protects the liver against injury induced by IR.

## Introduction

A definite cure for patients with hepatic cirrhosis can be organ transplant. Although this is a very useful strategy, it faces serious problems resulted from ischemia-reperfusion injury (Kurokawa et al., 1996 ▶). During IR injury, tissues do not receive enough oxygen and nutrients because of ceasing the blood flow (Kurokawa et al., 1996 ▶). Therefore, this event causes liver cell injury in two phases; in the first phase, ischemia, by disturbing the cell metabolism, and in the second phase, reperfusion, by producing and activating the apoptotic, and inflammatory factors as well as reactive oxygen species (ROS) (Klune and Tsung, 2010 ▶).

The physiological balance between oxidant and antioxidant (Paola et al., 2005 ▶) impairs following oxidative stress. It is well documented that the production and tissue levels of oxidants significantly increase after oxidative stress while at the same time, the tissue levels of antioxidant enzymes decrease (Sun et al., 2013 ▶).

Apoptosis activation after ischemia-reperfusion is considered one of the most important mechanism involved in tissue injury (Klune and Tsung, 2010 ▶). Caspase-3 is the last protein in both apoptosis pathways (cytoplasmic and mitochondrial) which damages tissues after activation. Antioxidants were shown to protect organs/tissues against oxidative stress by decreasing/inhibiting the mRNA expression of apoptotic factors. Conversely, increasing the expression level of caspase-3 leads to activation of apoptotic processes which in turn damage tissues (Porter and Janicke, 1999 ▶). IR injury was indicated to increase the serum level of caspase-3, therefore this apoptotic protein can activate the protease cascade and lead the cell to death (Contreras et al., 2004 ▶).

 Super oxide dismutase and catalase are two important enzymes that by scavenging intracellular ROS, protect cells against IR-induced damage (Yabe et al., 2001 ▶). Furthermore, the oxygen that separate from free radicals are one of the most important causes of cellular damage during IR injury in many organs such as the liver, intestine, and lung (Paola et al., 2005 ▶).

p-coumaric acid (PC) or trans-4-hydroxycinnamic acid is a phenolic compound obtained from plants in abundance. It is made of tyrosine by the enzymatic activity of tyrosine ammonia-lyase in the shikimic acid pathway. The time to maximum plasma concentration [tmax] and half-life [t1/2] for free, and conjugated form of p-coumaric are respectively 3.72-10 min and 15.9 min-1.3 h. PC is a safe compound because of having a very low toxicity (Its LD50 in mice is about 2850 mg/kg) and exerts its antioxidant activity through scavenging free radicals, and up-regulating the endogenous antioxidant enzymes. Moreover, evidence showed that PC by reducing apoptotic mediators and raising nuclear respiratory factors 1 (NRF-1) improves mitochondrial performance in stress situation (Guven et al., 2015 ▶; Mehta et al., 2012 ▶). The other reported beneficial effects of PC are anti-inflammatory, anti-hyperlipidemic, antimicrobial, anticancer, anti-diabetic, and neuroprotective effects and anti-coagulation activity (Abdel-Wahab et al., 2003 ▶; Amalan et al., 2016 ▶; Guven et al., 2015 ▶).

IR injury is a serious issue that can reject liver transplantation and it is the cause of many liver problems that clinically worth to prevent/decrease these consequences. Therefore, this study was performed to test hepatoprotective effect of p-coumaric acid on liver IR injury in rats.

## Materials and Methods

**Chemicals and kits**

p-coumaric acid, antioxidants kits, liver enzyme kits and anesthetics [ketamine+xylazine] were respectively purchased from Sigma- Aldrich (Merck KGaA, Darmstadt, Germany), Zellbio (GmbH, Germany), Pars Azmoon (Iran), and Alfasan (Woerden-Holland) companies.

**Animals**

Forty male Wistar rats purchased from Animal house of Ahvaz Jundishapur University of Medical Sciences weighing (170-220 g) were used in this study. They received rat's standard diet and were kept in normal conditions with room temperature (20-25°C), and 12/12 hr light/dark cycle. All experiments were done in accordance with ethical guidelines of Tehran Islamic Azad University, Science and research Unit (IR.IAU.SRB.REC.1397.130).

**Experimental groups and surgical procedures**

Animals were randomly assigned to the following groups: Normal (NC; intact group); Sham; p-coumaric acid (PC); IR-CO, and PC-IR. The time period of study was 7 days. Normal group did not experience any surgical procedure. This group received normal saline (2 ml/kg; intraperitoneally) for seven days and sacrificed on day 7. Rats in sham and IR-CO groups received 7% DMSO (as vehicle) diluted in normal saline (2 ml/kg; intraperitoneally) for seven consecutive days. Animals in PC and PC-IR groups received PC at 100 mg/kg for seven consecutive days with intraperitoneal injection (ip). On the 7^th^ day of the experiment, all groups received the last dose of normal saline, vehicle or p-coumaric acid. One hour later, all groups except NC, were anesthetized by an ip injection of a mixture of ketamine (60 mg/kg) + xylazine (15 mg/kg) and then underwent a midline laparotomy (Paola et al.). In addition, two groups of rats, PC-IR and IR, underwent IR injury (Mard et al.). To induce liver IR injury, hepatic triad (hepatic artery, portal vein and bile duct) was separated from adjacent tissues and then clamped by using a clamp artery to induce a partial ischemia (70%). Forty-five minutes after inducing ischemia, clamp was removed to establish reperfusion for one hour. At the end of reperfusion, rats were sacrificed by cardiac exsanguination and three pieces of their livers were removed, washed with N/S, and fixed in formalin solution for histopathology study. Two samples were snap-frozen in liquid nitrogen and kept at −80°C for molecular and enzymatic evaluations. 

**Determination of liver enzymes**

In order to determine the levels of ALT, AST, and ALP, blood was centrifuged at 3000 rpm for 10 min and serum was separated; then, serum concentrations of liver enzymes were quantified using commercial kits (Serum autoanalyser; BT-1500-A-A, Rome, Italy).

**Determination of antioxidants levels**

Liver homogenate was used to determine the levels of superoxide dismutase, glutathione (GSH), and catalase. In brief, livers were homogenated in proper volume of PBS, then centrifuged at 10000 rpm for 10 min. Supernatant was separated and used to determine the levels of studied antioxidant using Zell bio kits. 

**Determination of mRNA expression of caspase-3**

To quantify the changes of mRNA expression of caspase-3 following IR injury, the frozen tissues were homogenized in proper volume of lysis buffer RLT plus lysis buffer, and then total RNAs were extracted using RNeasy plus mini kit. A Nonadrop was used to determine the purity and concentration of the yielded RNA (NanodropThermo Scientific S.N:D015). One 1000 nanogram RNA was used to synthesize single stranded DNA (Quantitect Reverse Transcription Kit). The mRNA expression of targeted gene, caspase-3, and glyceraldehyde-3-phosphate as housekeeping gene was determined by semi-quantitative real-time PCR (qRT-PCR) using Roche Diagnostics 96^R^. The following primers were used to in PCR reaction: Caspase-3 (F: GGAACGAACGGACCTGTGG and R: CGGGTGCGGTAGAGTAAGC; and GAPDH, (F: TGCTGGTGCTGAGTATGTCGTG and R: CGGAGATGATGACCCTTTTGG). All the PCR reactions were done in a final volume of 20 μl (2 μl cDNA, 1 μl of each primers, 10 μl 2× real time Master Mix SYBR Green and 6 μl RNAse free water). Timing and temperature for RT-PCR reaction were: 95°C for 15 min for activating DNA Taq polymerase as initial incubation, followed by 40 cycles at 95°C for 20 sec, 51°C for 30 sec, and 72°C for 30 sec. A no-template negative control (H_2_O) was routinely run in every single PCR. The level of gene expression was normalized against GAPDH expression and 2^− ΔΔCt^ formula was used to determine the relative quantification of gene expression.

**Statistical analysis**

The data are presented as mean±standard error of means (SEM). One-way analysis of variance (ANOVA) followed by least significant difference test was used to analyze the data and a probability <0.05 considered significant. 

## Results

**Histological changes of the liver tissue following IR injury**

**Figure 1:**
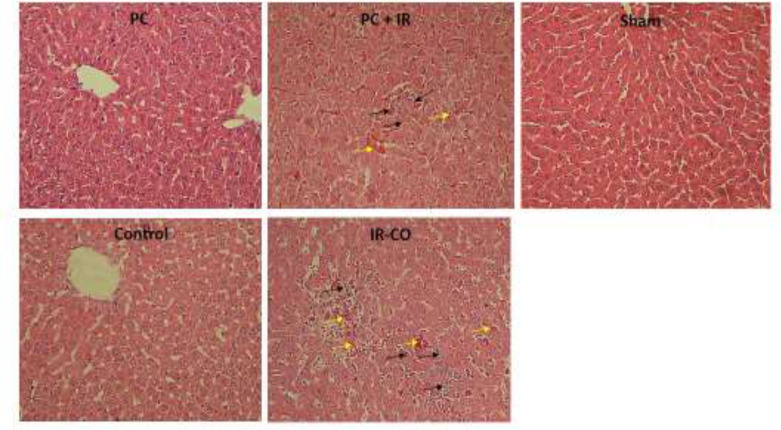
Microscopic representation of the liver tissue following IR injury (H&E staining ×400 magnification). Yellow and black arrows respectively show hyperemia and inflammation

The microscopic evaluation showed that the structure of liver in sham, normal, and PC groups was normal. This evaluation also showed severe histopathological changes including disruption of parenchymal regularity, congestion of RBCs, and infiltration of inflammatory cells in rats underwent hepatic ischemia-reperfusion. The pathological changes were mild in rats pretreated with p-coumaric acid. The liver tissue in PC-IR group demonstrated mild infiltration of inflammatory cells and congestion of RBCs (Figure 1). As shown in Table 1, liver inflammation and congestion of RBCs in IR-CO group were significantly increased in comparison to normal, PC, and Sham groups (p<0.001 in all cases). Pretreatment with PC significantly decreased histopathological changes of the liver in the PC-IR group compared to the IR group. 

**Liver enzyme levels following IR injury**

The serum level of ALT in rats underwent hepatic IR injury was significantly increased in comparison to the sham, PC, and normal group (p<0.001). 

**Figure 2:**
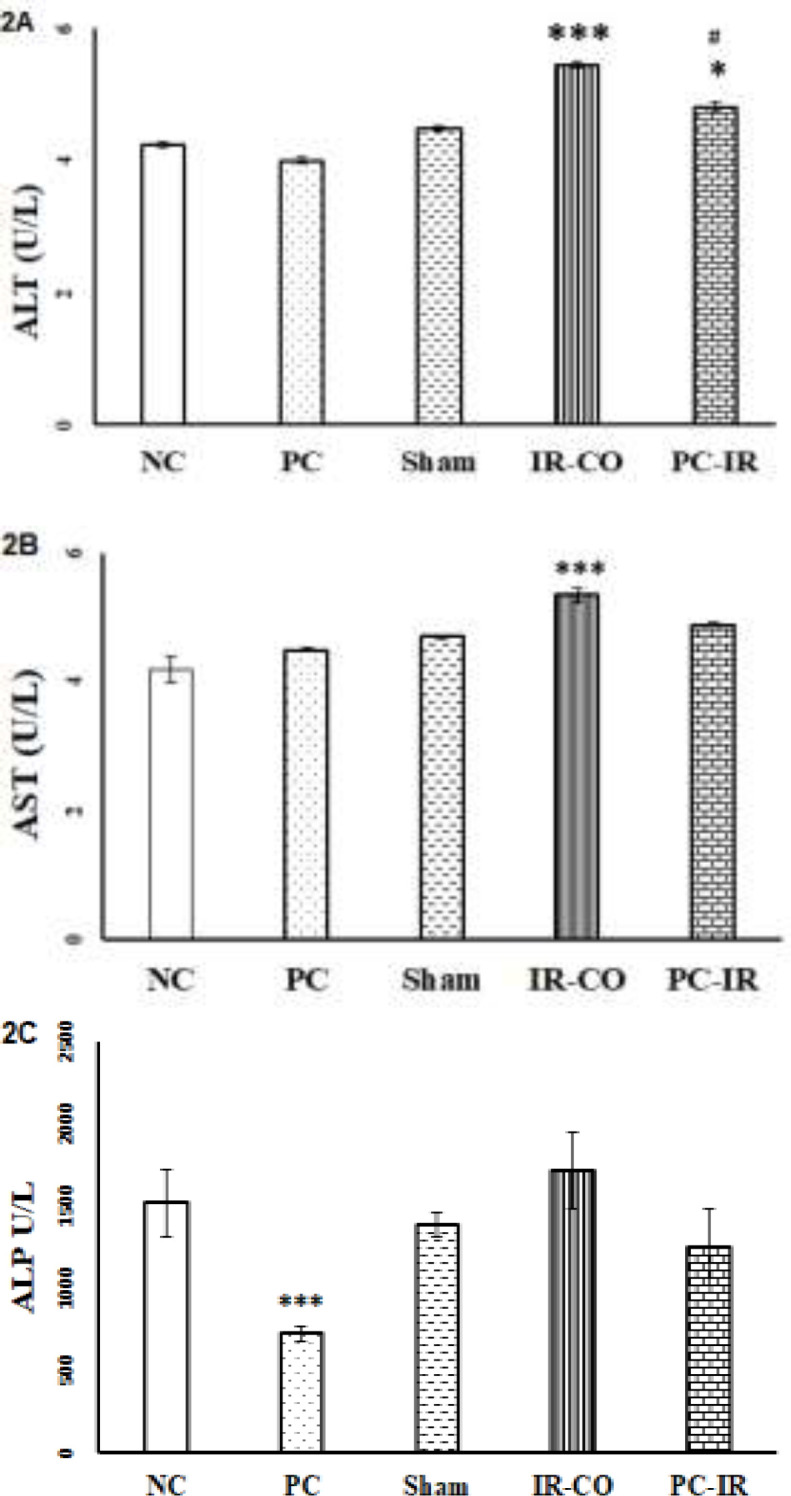
Effect of hepatic IR injury and p-coumaric pretreatment on ALT (2A), AST (2B), and ALP (2C) in male Wistar rats. Hepatic IR injury increased the levels of ALT, AST, and ALP while pretreatment with p-coumaric acid decreased these levels. NC (Normal intact group): these rats did not experience any surgical procedure. They received normal saline for seven days; Sham: Rats did not underwent hepatic IR injury. They received 7% DMSO (as vehicle) diluted in normal saline for seven days; PC and PC-IR groups: animals received p-coumaric acid (PC) at 100 mg/kg for the seven days; IR-CO and PC-IR: these groups underwent hepatic IR injury. ***p<0.001 versus sham, NC and PC groups. #p<0.01 compared to IR-CO rats and *p<0.05 versus sham. Data are presented as mean±S.E.M

Pretreatment with p-coumaric acid reduced ALT level to near normal (Figure 2A).The serum level of AST in IR-CO group was significantly increased in comparison to sham, PC, and normal groups (p<0.001). Pretreatment with p-coumaric acid reverted AST to normal level (Figure 2B). The results also demonstrated that ischemia followed by reperfusion non-significantly increased the serum level of ALP compared to the sham group (Figure 2C). Pretreatment with p-coumaric acid prevented any increase in the serum level of ALP in rats underwent IR injury. The lowest level of this enzyme was seen in PC rats.

**Table 1:** RBCs congestion and liver tissue inflammation

**Groups**	**Histological criteria**	
	**RBCs congestion**	**Inflammation**
PC	0.11±0.00	0.10±0.00
PC+IR	1.26± 0.23^***^#	1.23±0.34^***#^
Sham	0.14±0.03	0.13±0.02
NC	0.13±0.02	0.12±0.01
IR-CO	2.32± 0.36^***^	2.13±0.25^***^

***p<0.001 compared to NC and #p<0.001 versus IR-CO groups.

**Anti-oxidants levels following hepatic ischemia-reperfusion**

**Figure 3:**
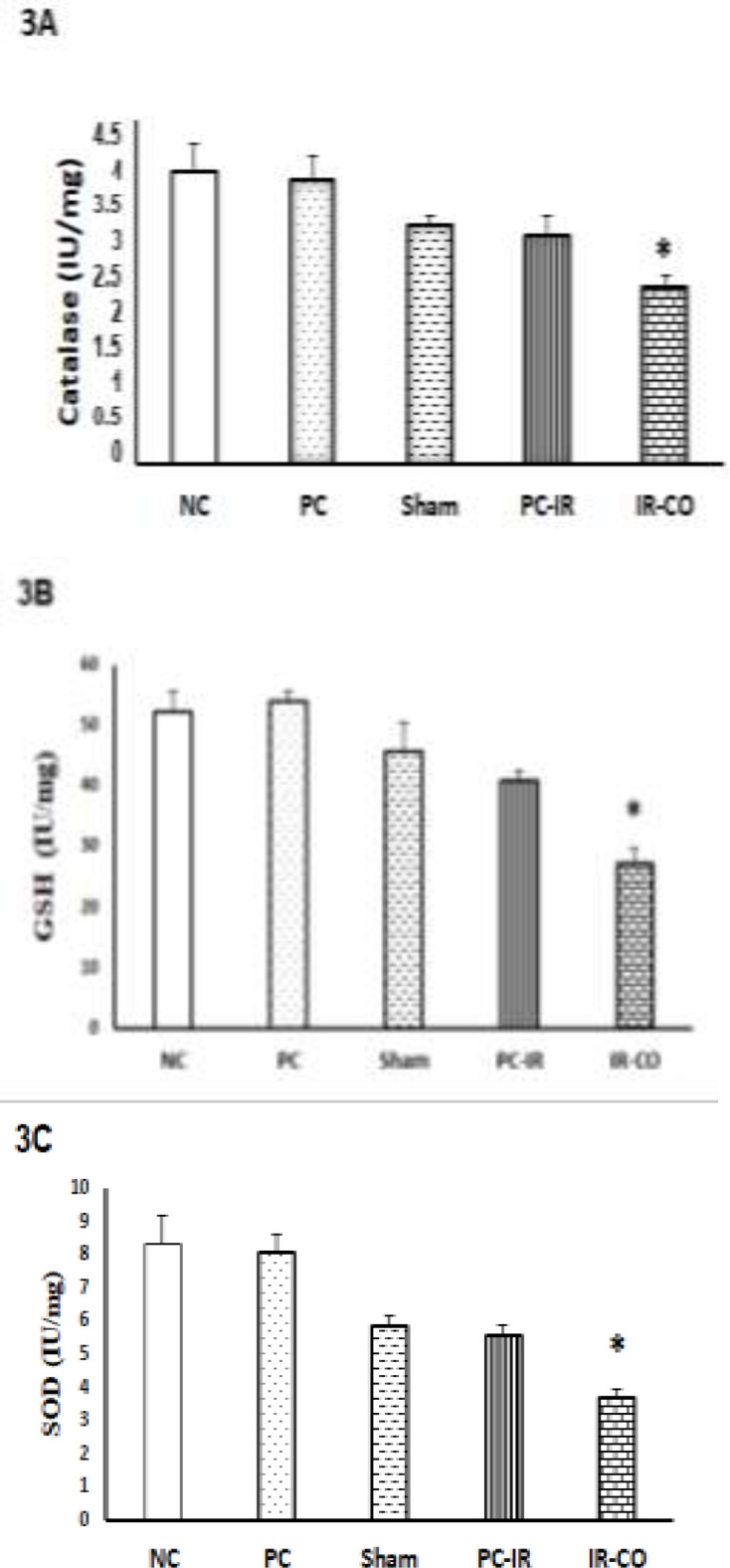
Effect of hepatic IR injury and p-coumaric pretreatment on catalase (3A), GSH (3B), and SOD (3C) in male Wistar rats. Hepatic IR injury decreased the levels of catalase, GSH, and SOD in liver tissue while pretreatment with p-coumaric acid increased these levels.*p<0.05 versus sham, NC, PC and PC-IR groups. Data are presented as mean±S.E.M

Forty-five ischemia followed by one hour reperfusion decreased the tissue levels of antioxidants (CAT, SOD, and GSH) (Figure 3A-C). These levels [CAT, SOD, and GSH] showed significant decreases compared to groups that did not experience IR including sham, PC, and NC groups (p<0.05, p<0.01 and p<0.001, respectively). Pretreatment with p-coumaric acid (100 mg/kg, i.p., seven consecutive days) restored these levels to sham levels in rats underwent IR injury. The highest levels of antioxidants (CAT, SOD, and GSH) were seen in the rats received p-coumaric acid (PC) and normal control groups. 

**The mRNA level of caspase-3 following ischemia-reperfusion in liver tissue**

**Figure 4:**
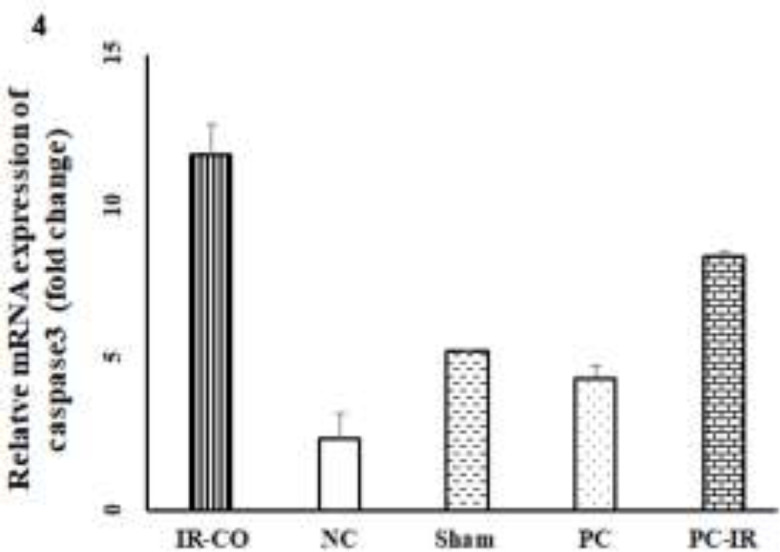
Effect of hepatic IR injury and p-coumaric acid pretreatment on caspase-3 mRNA expression level in male Wistar rats. Hepatic IR injury increased mRNA expression of caspase-3 in liver tissue while pretreatment with p-coumaric acid mitigated this increase. ***p<0.001 versus NC, PC, and PC groups. ^α^p<0.01 versus IR-CO group

Figure 4 shows that the mRNA expression of caspase-3 as an apoptosis indicator, significantly increased following hepatic IR injury. This level in IR-CO group was significantly higher compared to the sham, PC, and NC group (p<0.0001). Pretreatment with p-coumaric acid significantly but not completely, reduced this increase in PC-IR rats (p<0.05).

## Discussion

The present study showed that p-coumaric acid has hepatoprotective effect on IR injury. Pretreatment with p-coumaric acid for seven consecutive days before hepatic IR injury prevented pathological increase of AST and ALT in rats. This pretreatment also improved liver the antioxidant potency of liver tissue in rats underwent IR injury by increasing SOD, GSH, and CAT. 

The histopathological evaluation showed mild liver structural changes in rats received p-coumaric acid following liver IR injury. According to the current microscopic study, liver inflammation resulted following IR.

The disruption of parenchymal regularity, congestion of RBCs, and infiltration of inflammatory cells were apparent in rats underwent hepatic IR. These pathological changes were mild in PC pretreated rats. These outcomes showed that p-coumaric acid could mitigate the injury induced by hepatic IR. According to Table and Figure 1, pretreatment with p-coumaric acid at 100 mg/kg for seven consecutive days before inducing hepatic IR injury effectively but not completely decreased the adverse effects of IR injury. As the present results suggest, PC at the given dose alleviated harmful effects of IR up to 50%. Therefore, it seems that the studied dose or time of pretreatment probably were not enough to completely protect liver tissue from IR injury. This dose was selected according to previous reports which showed that PC at 100 mg/kg completely inhibited the adverse effect of IR injury (Ekinci Akdemir et al., 2017 ▶). The other reason for not achieving the complete protection of the liver can be due to the different rate of metabolism in organs. As a general fact, the liver is one the most metabolically active organs. Thus, higher doses or longer administration periods may be required to obtain complete protection by PC against injuries induced by IR. 

Liver amino transaminases (AST and ALT) and ALP over releases from hepatocytes to the systemic circulation and elevate in serum during and following the structural damage, necrosis, and cellular leakage (Aulbach and Amuzie, 2017 ▶). Our findings showed that liver IR injury significantly increased serum AST and ALT levels (p<0.001) while p-coumaric acid pretreatment decreased these levels compared to the IR control group (p<001). Previous studies showed that IR injury through increased ROS production and lipid peroxidation, disrupts integrity of cell membrane (Klune and Tsung, 2010 ▶), consequently liver enzymes enter the systemic circulation and elevated levels of them are achieved. Antioxidant agents such as crocin, and gallic acid, maintained integrity of cell membrane through lipid peroxidation inhibition and consequently improved AST and ALT serum levels (Akbari et al., 2017 ▶). Therefore, the above-mentioned reports along with current results about liver functional enzymes and results of microscopic evaluation mentioned earlier indicated that p-coumaric acid as an antioxidant via maintaining cell membrane integrity decreased AST and ALT release and improved their levels. 

In order to determine other possible mechanisms by which p-coumaric acid induced beneficial effect on IR injury, the mRNA expression of molecular marker for apoptosis (caspase-3) was also investigated. The corresponding result showed that this level in p-coumaric acid pretreated rats (PC-IR) was lower as compared to untreated rats, IR-CO rats. It was demonstrated p-coumaric acid protects rats heart against infarction–induced by isoproterenol through mitigating apoptosis (Stanely Mainzen Prince and Roy, ▶ 2013). Another study also showed that gallic acid and p-coumaric acid by exerting anti-apoptotic activity induce their neuroprotective activity in rats hippocampus in diabetic-induced neurodegeneration (Abdel-Moneim et al., 2017 ▶). Therefore, the present result was in agreement with the above-mentioned reports which concluded that the hepatoprotective effect of p-coumaric acid on IR injury is partly mediated by decreasing the gene expression of caspase-3.

A study showed that p-coumaric acid pretreatment has neuroprotective effect in cerebral ischemia-reperfusion and increased levels of SOD and catalase (Sakamula and Thong-Asa, 2018 ▶).

 Others reports showed that p-coumaric acid can protect the liver against hepatotoxicity induced by cisplatin, through increasing the level of antioxidants such as SOD and GSH (Ekinci Akdemir et al., 2017 ▶). The present results also showed that the activity of all studied antioxidant enzymes such as SOD and CAT, as well as tissue level of GSH increased in rats pretreated with p-coumaric acid. This finding suggested that p-coumaric acid pretreatment enhanced liver free radical scavenging ability by increasing liver antioxidants (SOD, CAT, and GSH). What is the clinical importance of these results? Pretreatment with p-coumaric acid can help patients suffering from end stage liver diseases who are candidate for transplant (Hassan et al., 2005 ▶).

These findings showed that p-coumaric acid through improving the antioxidant levels (SOD, CAT and GSH) of liver tissue, reducing liver functional tests (AST, ALT, and ALP) and down-regulating apoptotic protein, caspase-3, protected this organ against injury induced by IR in rats. 
